# Supplementary material for: Transcription and translation contribute to gene locus relocation to the nucleoid periphery in E. coli
Source: Nat Commun. 2019 Nov 12;10:5131. doi: 10.1038/s41467-019-13152-y (PMC6851099; doi:10.1038/s41467-019-13152-y)
Supplement: Supplementary file 1 — Supplementary lnformation [file 41467_2019_13152_MOESM1_ESM.pdf]

Supplementary Information for

"Transcription and translation contribute to gene locus relocation to the nucleoid periphery in *E. coli*"

Sora Yang[1,†], Seunghyeon Kim[2,†], Dong-Kyun Kim[3], Hyeong Jeon An[2], Jung Bae Son[1], Arvid Hedén Gynnå[4], and Nam Ki Lee[1,*]

[1] Department of Chemistry, Seoul National University, Seoul 08826, Korea

[2] Department of Physics, Pohang University of Science and Technology, Pohang 790-784, Korea

[3] School of Interdisciplinary Bioscience and Bioengineering, Pohang University of Science and Technology, Pohang 790-784, Korea

[4] Department of Cell and Molecular Biology, Uppsala University, Uppsala 75236, Sweden

[†] These authors contributed equally to this work.

[*] To whom correspondence should be addressed. E-mail: namkilee@snu.ac.kr

Supplementary Notes

Supplementary Note 1. Model of the transcriptional kinetics

To determine transcriptional on-rate (k_{on}) and off-rate (k_{off}, *i.e.*, elongation rate), we fit the data of the average number of transcribing T7 RNAP per cell (Fig. 1e) to an exponential equation derived from the simple kinetic model in Fig. 1d as follows:

$$\frac{d\langle \text{RNAP}_{\text{transcribing}}\rangle}{dt} = k'_{\text{on}}\langle \text{RNAP}_{\text{diffusing}}\rangle - k_{\text{off}}\langle \text{RNAP}_{\text{transcribing}}\rangle \quad (1)$$

Considering there are approximately 35 RNAPs in diffusing state, while there is less than one in transcribing state in our measurement conditions, $\text{RNAP}_{\text{diffusing}} = \text{RNAP}_{\text{total}} - \text{RNAP}_{\text{transcribing}} \approx \text{RNAP}_{\text{total}}$, where $\text{RNAP}_{\text{total}}$ is a constant. Using the initial condition of $\text{RNAP}_{\text{transcribing}} = 0$ at t = 0, the solution equation is given by

$$\langle \text{RNAP}_{\text{transcribing}}\rangle = \frac{k'_{\text{on}}\langle \text{RNAP}_{\text{total}}\rangle}{k_{\text{off}}}\left(1 - e^{-k_{\text{off}}t}\right) \quad (2)$$

Because the termination time at the termination site, typically less a few seconds, is much smaller than that of the elongation duration time (tens of seconds), the inverse of k_{off} corresponds to the elongation duration time from the promoter to the termination site. Thus, we can calculate the elongation rate from $L_{\text{gene-length}}\cdot k_{\text{off}}$., where $L_{\text{gene-length}}$ is the length of gene. The transcriptional on-rate, $k_{\text{on}} = k'_{\text{on}}\langle \text{RNAP}_{\text{total}}\rangle$, is determined from $k'_{\text{on}}\langle \text{RNAP}_{\text{total}}\rangle / k_{\text{off}}$. The average number of T7 RNAP in cells, $\langle \text{RNAP}_{\text{total}}\rangle$, was obtained from the integrated fluorescence intensity from each cells (Supplementary Fig. 1d.). We defined $\langle \text{RNAP}_{\text{total}}\rangle$ as the mean values of the histogram, shown in Supplementary Fig. 1e, divided by $\langle I_{\text{single}}\rangle$ from the single eYFP intensity obtained from Supplementary Fig. 1c

Supplementary Note 2. Simulation of gene movement

The mean localization error in Fig. 2a is approximately 70 nm, obtained from $\sigma = \sqrt{\frac{s^2}{N} + \frac{a^2}{12N} + \frac{8\pi s^4 b^2}{a^2 N^2}}$, where ***s*** is the standard deviation of the Gaussian distribution, ***a*** is the pixel size, ***b*** is the background noise, and ***N*** is the number of collected photons. In this condition, the difference of the average localization between two transcription distributions can be distinguished down to approximately 21 nm when the number of averaging dots is more than 1000, as the error of the mean position decreases inverse proportionally to the square root of the number of dots. To confirm whether the average position difference, smaller than the localization error (70 nm), indicates bona-fide gene movement, we performed a simulation for the localization movement. First, 100,000 random spots were generated in the three-dimensional coordinates (x_i, y_i, z_i) under the conditions for which are as follows: (1) -0.35 < x_i, y_i < 0.35, (2) -1 < z_i < 1, and (3) the radial distance ($r = \sqrt{x_i^2 + y_i^2}$) must be less than 0.35 for mimicking a nucleoid condensation (in our localization analysis, we set the short axis of a cell for 1) (Fig. 2e, blue dots).

Each dot was moved in the radial direction by distance ***L***(*j*) (*j* = 1, 2, 3, ···, 100000), where the distribution of ***L*** followed an exponential distribution by assuming that the distance of each dot moved is a random variable with a constant probability. The distribution of ***L*** was generated to have a specific mean value $L_{\text{x\_mean}}$ for its x component, $< |L\cos\theta| >= L_{\text{x\_mean}}$ (where *θ* is the radial angle of the spot), to mimic the spatial difference of the experimental value. Normally distributed noise was added for localization error to each initial coordinates (x_i, y_i, z_i) and final coordinates (x_f, y_f, z_f) after moving L distance (Fig. 2e, red dots), which resulted in (Dx_i, Dy_i, Dz_i) and (Dx_f, Dy_f, Dz_f), respectively. While the initial coordinates (x_i, y_i, z_i) were limited in the radial distance $r < 0.35$, the coordinates detected after adding the localization error (Dx_i, Dy_i, Dz_{ii}) could be localized at radial distance $r \geq 0.35$ due to the localization error as shown in Fig. 2f. The distribution plotted in Fig. 2g (cyan bar) was generated with $L_{\text{x\_mean}} = 0.07$ in the relative x-axis, which corresponds to 61 nm in three-dimensional geometry, matching the difference of average position between the initial (< 50 sec) and the final conditions (300 sec - 350 sec) obtained from the experimental results (Fig. 2c-d). The distributions of simulated spots were similar with the experimental results (Fig. 2f-g).

While the detection noise was larger than the moving distance *L*, increasing the number of averaged spots reduced the variance of average displacement small enough to verify the difference. Supplementary Fig. 4b shows that the standard deviation of the mean position is inversely proportional to the square root of the number of dots. To confirm that the averages for two populations were statically different from each other, a two-tailed *t*-test was used to judge the difference between their means

relative to the variability of their positions. The probability of a rejection of the null hypothesis at the 5% significance level was obtained from repeating t-test 1000 as the number of selected spots increases (Supplementary Fig. 4c). As the number increased more than 300, two populations were significantly different from one another with 99.9% probability. To find the smallest distinguishable difference, t-test was used by changing the moving distance from 1 to 70 nm for the selected spot number, N = 350 and 1500 (Supplementary Fig. 4d). Approximately 40 nm difference in the three-dimensional geometry could be distinguished with over 95% probability by averaging 350 spots, and for 1500 spots the value decreased to 19 nm.

Supplementary Figures

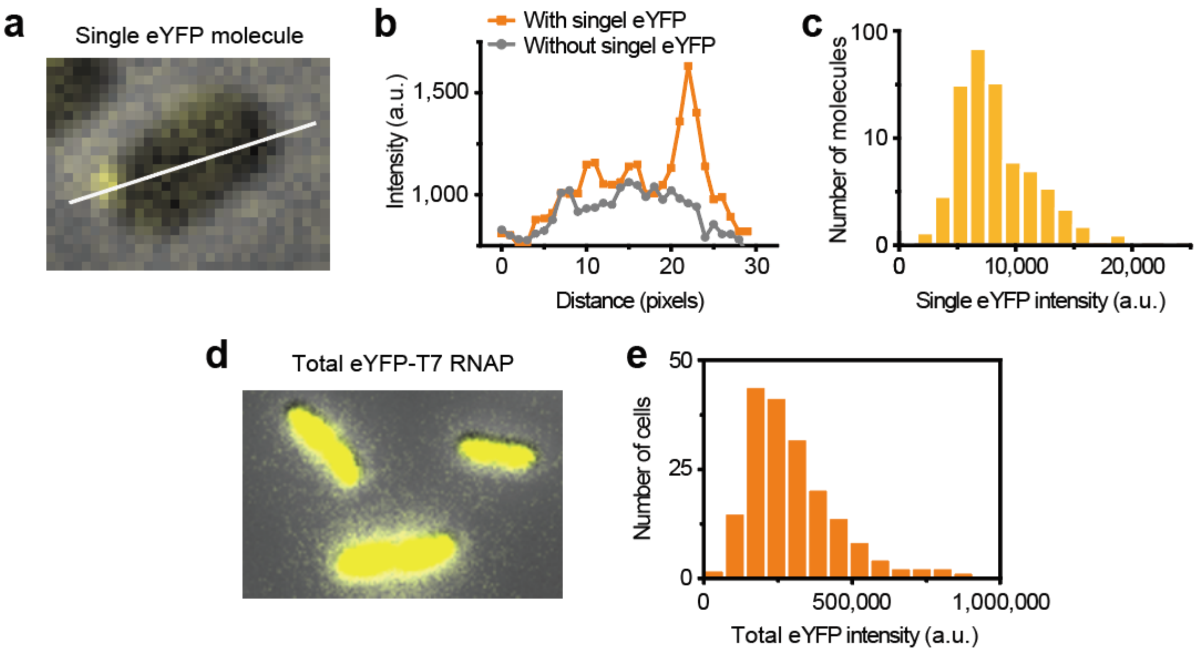

Supplementary Figure 1. Measurement of the fluorescence intensity of a single eYFP and the number of eYFP-T7 RNAP in a cell.

(a) Detection of single eYFP molecule using Tsr-eYFP (strain NL003). A single Tsr-eYFP molecule was detected as a diffraction-limited spot.

(b) Line-scan of fluorescence intensity from the white line in (a). Single-step photobleaching was used to confirm a single Tsr-eYFP molecule.

(c) Distribution of fluorescence intensity of single Tsr-eYFP. The mean value of the distribution was used to estimate the total number of eYFP-T7 RNAP in a cell.

(d) Overlay images of *E. coli* cells (T7p_4.5kb) obtained by phase contrast and fluorescence images.

(e) Histogram of total fluorescence intensity of cells. The average total number of eYFP-T7 RNAP molecules per cell was obtained using the single Tsr-eYFP intensity obtained from (c).

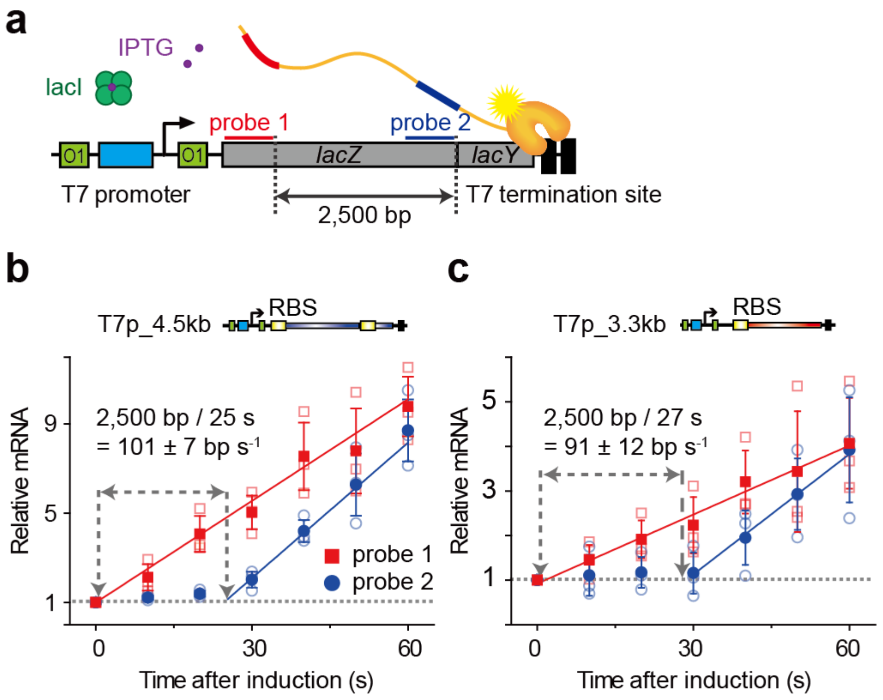

Supplementary Figure 2. Measurement of *in vivo* transcription elongation rate using real-time RT-PCR.

(a) A schematic of real-time RT-PCR design for the measurement of RNAP's elongation rate. Two taqman probes were located within the *lacZ* gene. The distance between the two probes was 2,500 bp. (b-c) The relative expression level of mRNA after 1 mM IPTG induction was detected by real-time RT-PCR. The time RNAP arrives at each probe region was measured by the linear fit of the increase of mRNA level (red line for probe 1 and blue line for probe 2). The elongation rate was calculated by dividing the distance between the two probe regions (2,500 bp) by the time between probe 1 and probe 2 (indicated by arrows). The elongation rates of T7p_4.5kb and T7p_3.3kb were measured to be 101 ± 7 bp s^{-1} (b) and 91 ± 12 bp s^{-1} (c), respectively. All error bars were s.d. from three independent experiments at the room temperature using the same growth conditions used for the image measurement in Fig. 1.

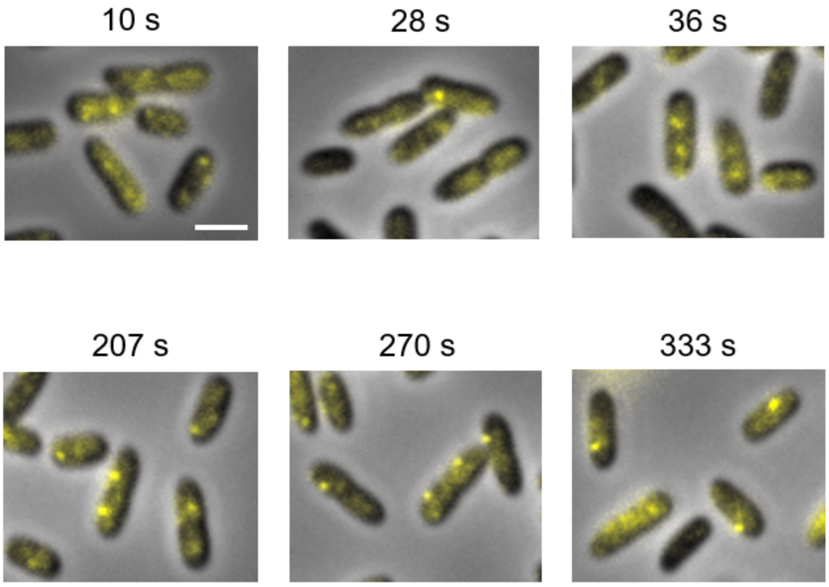

Supplementary Figure 3. Additional representative images of T7p_4.5kb cells after adding IPTG. When the image was obtained relative to transcription induction by IPTG is specified in each image. The images obtained at early times showed diffraction-limited spots localized close to the center of the cells, but few spots were observed. After 200 sec of IPTG induction, diffraction-limited spots localized close to the plasma membrane were observed more frequently. Scale bar, 2 µm.

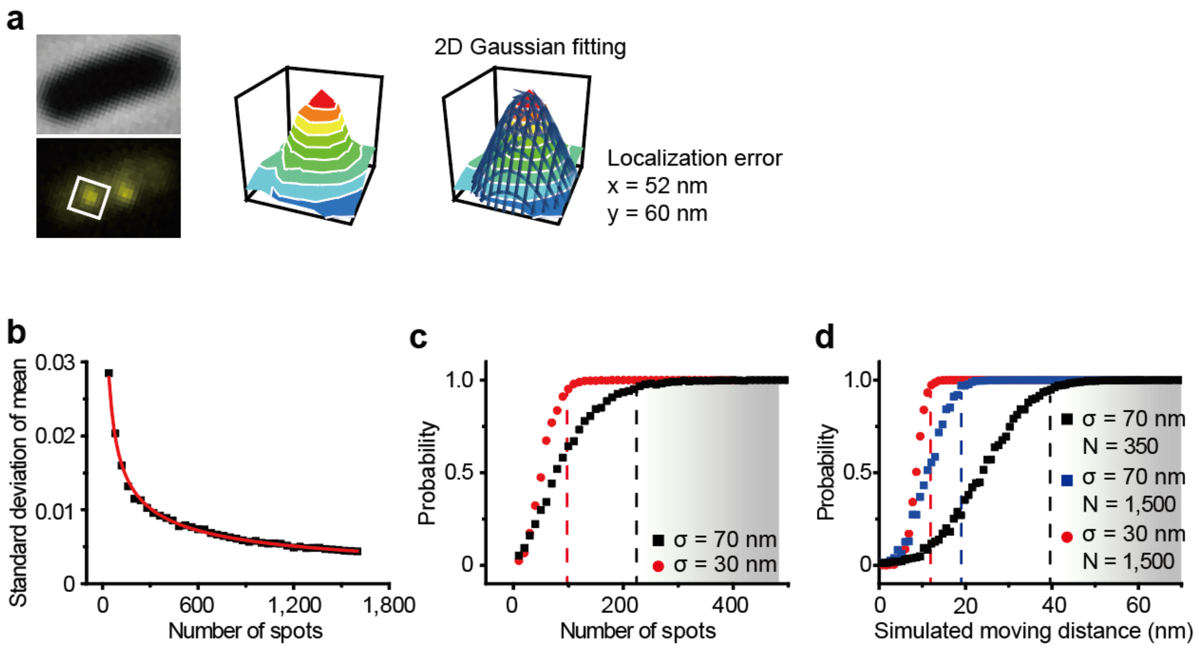

Supplementary Figure 4. Localization error of the transcription spots and the simulation of the standard error of the mean position of diffraction-limited spots.

(a) Left panel, phase contrast and fluorescence images of T7p_4.5kb. Middle panel, fluorescence intensity contour of a transcription spot selected from the fluorescence image (the white square). Right panel, two-dimensional Gaussian fitting of the transcription spot. Typical localization error was 70 nm.

(b) The standard deviation of the mean position of the selected spots in x-axis is plotted as a function of number of selected spots. The fitting curve is $a/\sqrt{N}$, where $\boldsymbol{a}$ is the standard deviation of the whole spots and $\boldsymbol{N}$ is the number of spots.

(c) The probability of a rejection of the null hypothesis to identify the number of spots needed for distinguishing the movement of fluorescent spots. The probability is plotted as a function of the number of fluorescent spots measured, where the average moving distance L_{x_mean} was set as 0.07 (61 nm in three dimensional coordinate) and the significance level was 5%. Dotted lines indicate 95% probability, where the number of fluorescent spot was 220 (the localization error, σ = 70 nm) and 100 (σ = 30 nm). These simulation results indicate that 0.07 movement in our measurement could be distinguished if the number of fluorescent spot is more than 220 (σ = 70 nm) and 100 (σ = 30 nm). The t-test was repeated 10,000 times.

(d) The probability of rejection of the null hypothesis depending on the moving distance of fluorescent dots. The probability is plotted as a function of average moving distance L_{x_mean} at the 5% significance level. Black squares, blue squares and red circles denote the probability when the number of averaged spots were 350, 1500 (σ = 70 nm) and 1500 (σ = 30 nm), respectively. As the number of averaged spots increased, the distance limit, distinguishable in our measurement, was lowered from 40 nm in 350 spots to 19 nm in 1500 spots for 95% probability (σ = 70 nm) (dotted lines). The distance

limit was 12 nm when the number of average spots were 1500 (σ = 30 nm). The t-test was repeated 10,000 times.

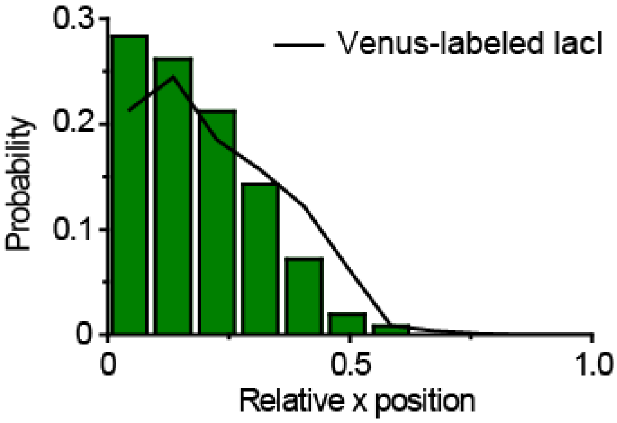

Supplementary Figure 5. Comparison of the localization of *lacZ* gene locus at the initial stage of transcription with that of venus-labeled LacI.

The localization of venus-labeled LacI denotes the location of *lacZ* gene under repressed condition. The distribution of transcribing genes at the initial stage, which appear as fluorescent spots before 50 sec after induction, is very similar with that of venus-labeled LacI (black line) (from 810 spots).

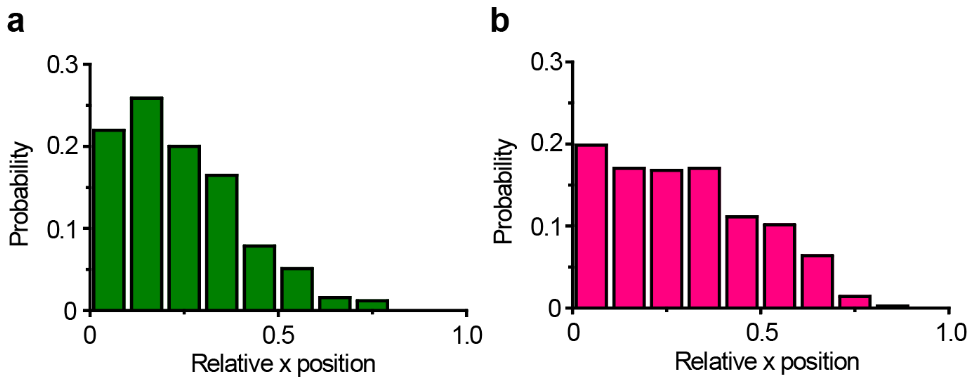

Supplementary Figure 6. The distribution of gene locus localizations transcribed by eYFP-T7 RNAP for T7p_3.3kb strain.

(a) Initial localization of gene locus of strain T7p_3.3.kb, until 50 sec after induction.

(b) The localization of the gene locus between 300 sec and 350 sec after IPTG induction, which indicates the movement of gene locus outside nucleoid.

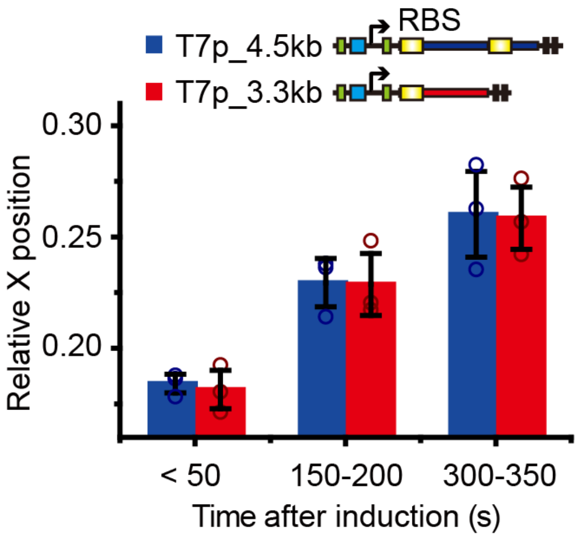

Supplementary Figure 7. Quantitative gene locus movement analysis of strains T7p_4.5kb (blue bar) and T7p_3.3kb (red bar).

Average relative x-positions of the genes transcribed by eYFP-T7 RNAP within each time window are shown. All error bars were s.d. from three independent experiments.

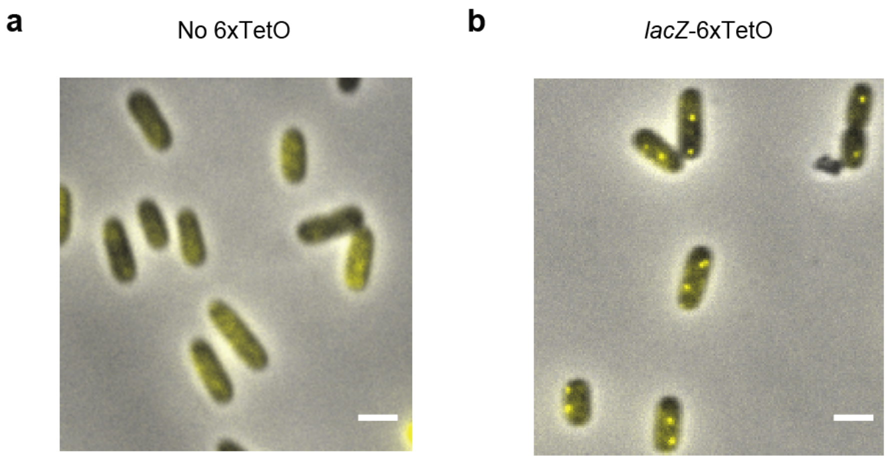

Supplementary Figure 8. The localization of TetR-eYFP was not observed without 6xTetO array in the genome. Fluorescence-phase contrast overlay images.

(a) No-diffraction limited spot of TetR-eYFP was detected without 6xTetO array in the genome. A blurred eYFP fluorescence signals from the diffusing TetR-eYFPs were detected in whole cell region.

(b) Diffraction limited spots were detected in the strain *lacZ*-6xTetO using the same experimental condition as in (a). The spots indicate the localization of 6xTetO array on the downstream of *lacZ* gene. Scale bar, 2 µm.

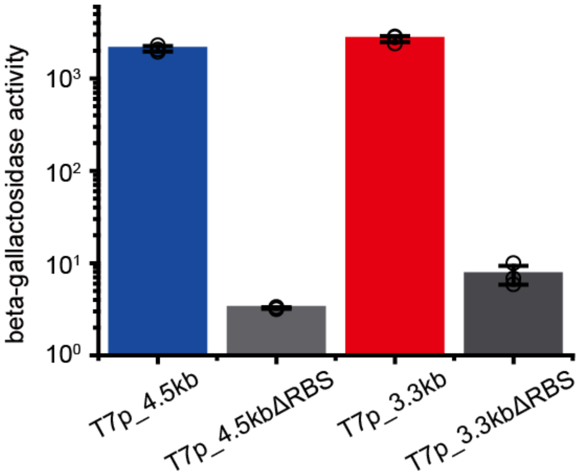

Supplementary Figure 9. Miller assay. Miller assay shows that the LacZ expression was reduced by a factor of 646 for T7p_4.5kb and 355 for T7p_3.3kb, when the *lacZ* RBS sequence (AGGAGG) in the wild type lac operon was replaced with the random sequence (CTCGAG). All error bars were s.d. from three independent experiments.

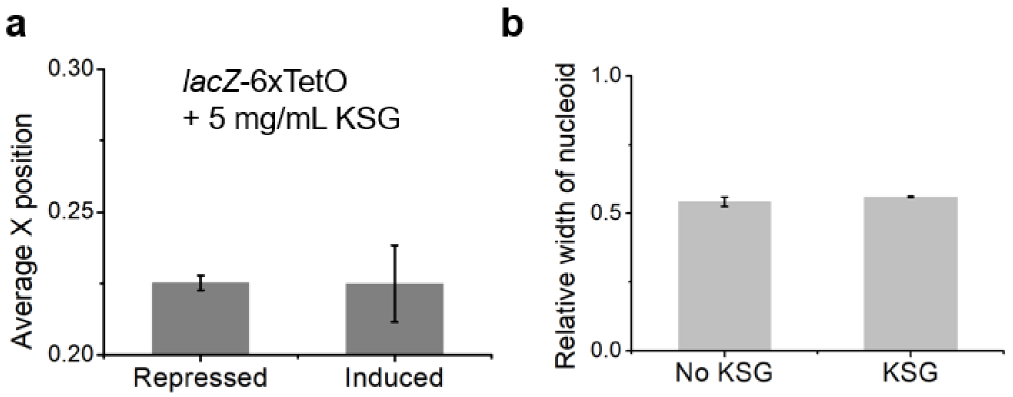

Supplementary Figure 10. Effect of kasugamycin (KSG) on the movement of gene loci by *E. coli* RNAP-driven transcription.

(a) Average relative x-positions of gene loci were obtained in cells treated with 1 mM IPTG (induced) and without IPTG (repressed) at 15 min after 5 mg/ml kasugamycin treatment to the *lacZ*-6×TetO strain. The experiments were performed at 24 degrees.

(b) The relative width of the nucleoid compared to the cell width with and without KSG treatment. The error bars were obtained from three independent experiments (s.d.).

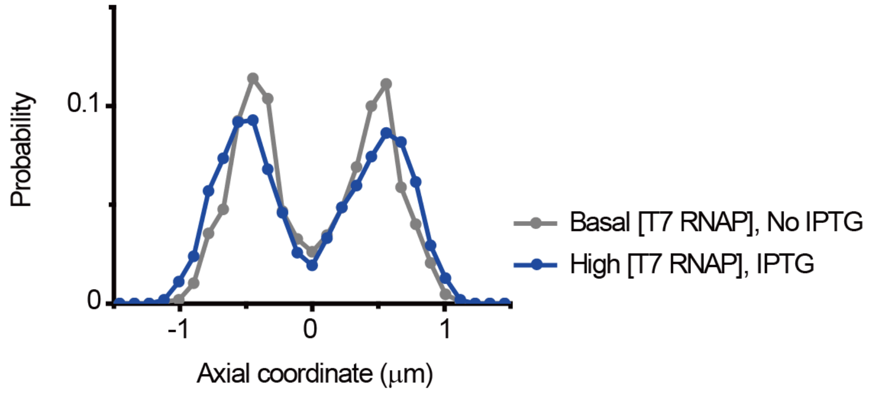

Supplementary Figure 11. Distributions of the locations of the *lacZ* gene locus along the axial coordinate (longer length of *E. coli* cell) for T7p_*lacZ*-12xTetO strain in Fig. 5a. The gray line denotes the distribution of the gene locus with a basal level of T7 RNAP and no IPTG. The transcription by T7 RNAP induced the movement of the gene locus outwardly in the axial direction by 78 ± 32 nm.

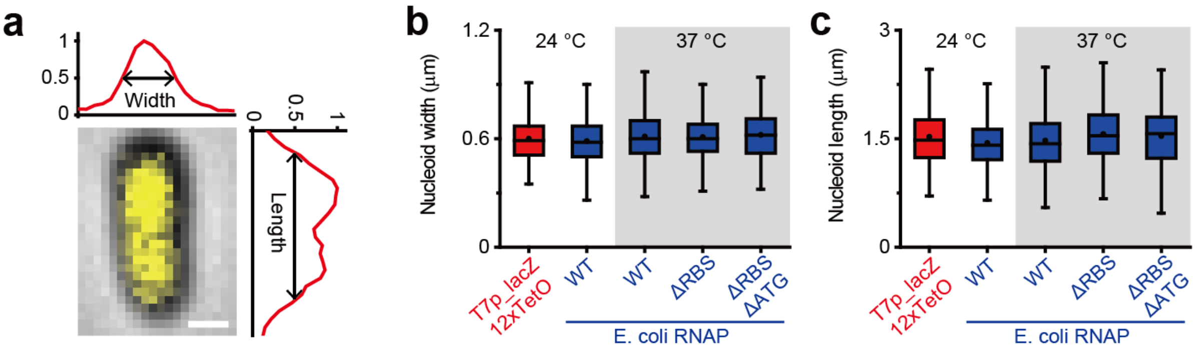

Supplementary Figure 12. Nucleoid imaging using SYTOX-Green dye.

SYTOX-Green dye (Invitrogen #S7020) was added to the cell culture at a final concentration of 500 nM and the cells were grown by shaking for 1 hour. Before imaging, the cells were washed twice to remove free dyes. The nucleoid width and length were defined as the full width at half maximum of the intensity profiles of SYTOX fluorescent images, along the short and long axes of the cell, respectively. (a) A representative cell shown in a phase contrast overlaid with SYTOX fluorescent image (Scale bar, 500 nm). The nucleoid width and length were defined as the full width at half maximum of the intensity profile along the short and long axes of the cell, respectively.

(b-c) Comparison of the nucleoid (b) width and (c) length in each experimental condition. The nucleoids were maintained at a similar size (width = 0.6 ± 0.01 μm and length = 1.53 ± 0.04 μm) for both T7 RNAP- and *E. coli* RNAP-mediated transcriptions. (For each box, center spot, mean; center line, median; box limits, 25th and 75th percentiles; whiskers, 1.5x interquartile range)

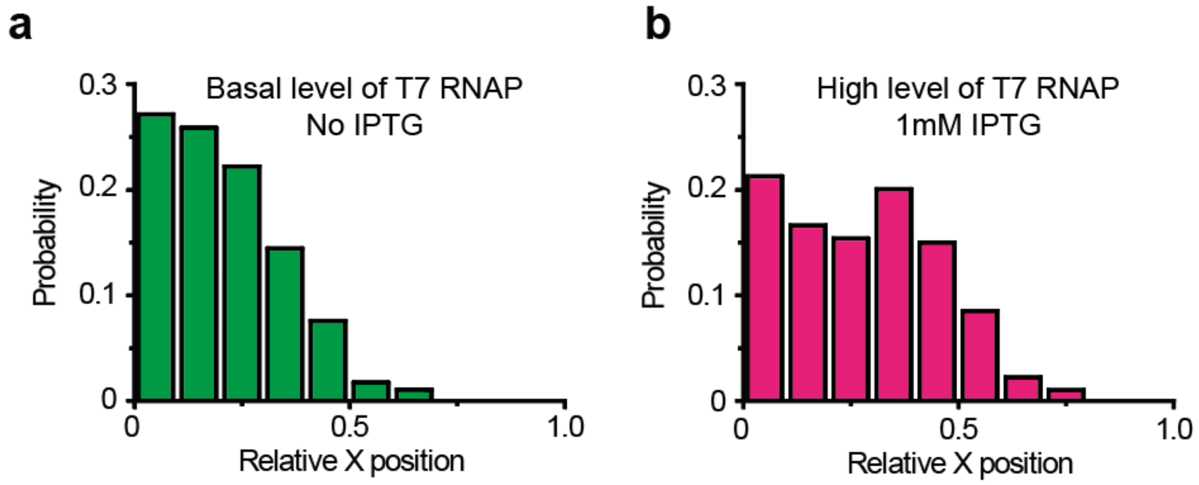

Supplementary Figure 13. Effect of translation initiation blocking by antibiotics on gene locus movement. The distributions of the subcellular localization of gene locus transcribed by T7 RNAP after 15 min incubation with 5 mg ml^{-1} kasugamycin (translation initiation inhibitor).

(a) The distribution of gene locus with the basal level of T7 RNAP (no rhamnose) and no IPTG.

(b) The distribution of gene locus when T7 RNAP was expressed using 0.2 % L-rhamnose and 1mM IPTG was added.

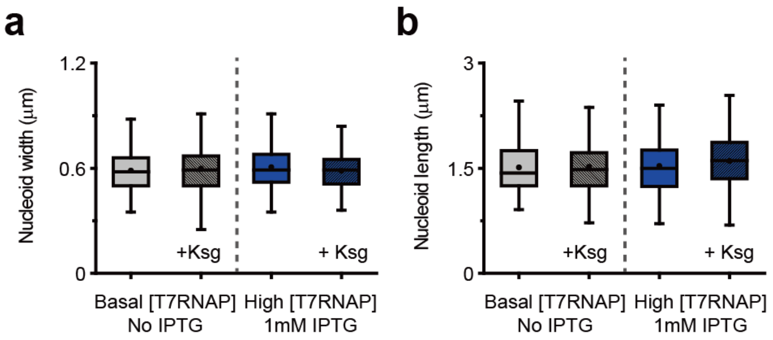

Supplementary Figure 14. Comparison of the nucleoid (a) width and (b) length with and without 5 mg ml^{-1} Ksg treatment (after 15 min incubation). (For each box, center spot, mean; center line, median; box limits, 25th and 75th percentiles; whiskers, 1.5x interquartile range)

a

Probe 1 Ct	(+ RBS)	19.7 ± 0.2
Probe 1 Ct	(- RBS)	24.9 ± 0.3
Relative mRNA quantity (+ RBS / - RBS)		36

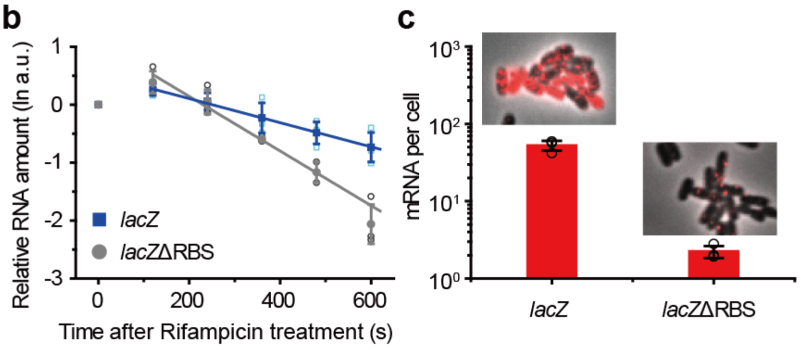

Supplementary Figure 15. Measurement of total mRNA expression level and the degradation rate of *lacZ* mRNA.

(a) Comparison of the relative *lacZ* mRNA quantity expressed by *E. coli* RNAP in the presence and absence of RBS using real-time RT-PCR. LacZ mRNA with RBS was expressed 36-fold more compared with the RBS-deleted strain at the steady state. Data are shown as mean values ± standard deviations obtained from three independent experiments.

(b) Measurement of the degradation rate of mRNAs. Rifampicin, blocking transcription initiation of *E. coli* RNAP, is typically used for measuring mRNA degradation rate. At time zero, rifampicin was added to stop transcription initiation. Using real-time RT-PCR, the mRNA abundance was measured by quantitative RT-PCR at multiple time points. The half-life of the *lacZ* transcript was 5.5 ± 0.8 min and of the *lacZ*ΔRBS transcript 2.4 ± 0.3 min. Error bars indicate the s.d. of three independent experiments.

(c) The mean number of mRNA per cell measured by single-molecule FISH (smFISH). The strain expressed 24 times more mRNAs in the presence of RBS at the steady state. Error bars represent the s.d. of three independent experiments.

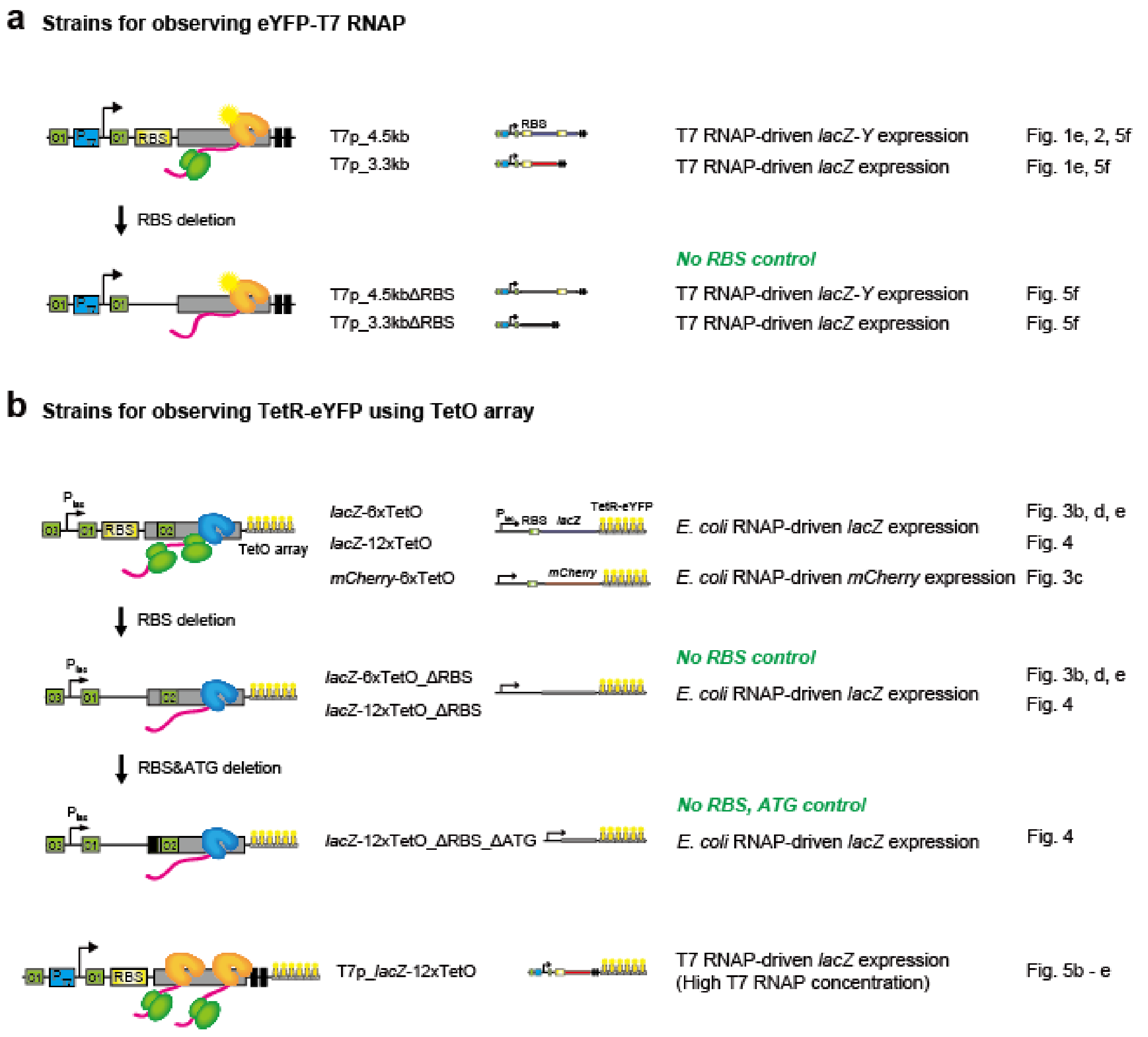

Supplementary Figure 16. Summary of strains.

(a) The strains for observing eYFP-T7 RNAP transcribing downstream of the T7 promoter. T7p_4.5kb; T7 RNAP-driven *lacZ-Y* expression, used in Fig. 1e, 2, and 5f. T7p_3.3kb; T7 RNAP-driven *lacZ* expression, used in Fig. 1e and 5f. RBS-deleted strains were used in Fig. 5f.

(b) The strains for observing TetR-eYFP bound to the TetO array inserted downstream of a specific gene. *lacZ*-6xTetO; *E. coli* RNAP-driven *lacZ* expression, used in Fig. 3b, d, and e. *lacZ*-12xTetO; *E. coli* RNAP-driven *lacZ* expression, used in Fig. 4. *mCherry*-6xTetO; *E. coli* RNAP-driven *mCherry* expression, used in Fig. 3c. *lacZ*-6xTetO_ΔRBS; the RBS-deleted strain from *lacZ*-6xTetO was used in Fig. 3b, d, and e. *lacZ*-12xTetO_ΔRBS; the RBS-deleted strain from *lacZ*-12xTetO was used in Fig. 4. *lacZ*-12xTetO_ΔRBS_ΔATG; the start codon was mutated (ATG→TAA) from *lacZ*-12xTetO_ΔRBS and this strain was used in Fig. 4. T7p_*lacZ*-12xTetO; T7 RNAP-driven *lacZ* expression, used in Fig. 5b-e for the high T7 RNAP concentration experiments.

Supplementary Table 1. Primers and probes used for real-time RT-PCR measurement

Primer	Sequence (5’ – 3’)
N fwd	GAAGGCCAGACGCGAATTATTTT
N rev	GACTGTCCTGGCCGTAACC
N probe	(6-FAM)ACTCGGCGTTTCATCTG(MGB)
C fwd	TCAACAGCAACTGATGGAAACCA
C rev	GATATTCAGCCATGTGCCTTCTTC
C probe	(6-FAM)CCATCGCCATCTGCTG(MGB)
