# Supplementary material for: Transcription and translation contribute to gene locus relocation to the nucleoid periphery in E. coli
Source: Nat Commun. 2019 Nov 12;10:5131. doi: 10.1038/s41467-019-13152-y (PMC6851099; doi:10.1038/s41467-019-13152-y)
Supplement: Supplementary file 3 — Description of Additional Supplementary Files [file 41467_2019_13152_MOESM3_ESM.pdf]

Description of Additional Supplementary Files

File Name: Supplementary Movie 1
Description: Representative live-cell time-lapse images of *lacZ* gene locus movement after IPTG induction at 24 degrees. Twelve repeat of tetO was inserted on the downstream of *lacZ* gene transcribed by *E. coli* RNAP. The TetR-eYFPs bound to the tetO array were detected as fluorescence spots. Time in min:sec. Scale bar, 1.5 µm.

File Name: Supplementary Movie 2
Description: Additional representative live-cell time-lapse images of *lacZ* gene locus movement after IPTG induction, measured at the same experimental condition of Supplementary Movie 1. Time in min:sec. Scale bar, 1.5 µm.
